# A machine learning approach to estimating preterm infants survival: development of the Preterm Infants Survival Assessment (PISA) predictor

**DOI:** 10.1038/s41598-018-31920-6

**Published:** 2018-09-13

**Authors:** Marco Podda, Davide Bacciu, Alessio Micheli, Roberto Bellù, Giulia Placidi, Luigi Gagliardi

**Affiliations:** 1 Dipartimento di Informatica, Università di Pisa, Pisa, Italy; 2 Terapia Intensiva Neonatale, Ospedale A. Manzoni, Lecco, Italy; 3 Italian Neonatal Network, Meda, Italy; 4 Pediatrics and Neonatology Division, Ospedale Versilia, Viareggio, AUSL Toscana Nord Ovest, Pisa, Italy

## Abstract

Estimation of mortality risk of very preterm neonates is carried out in clinical and research settings. We aimed at elaborating a prediction tool using machine learning methods. We developed models on a cohort of 23747 neonates <30 weeks gestational age, or <1501 g birth weight, enrolled in the Italian Neonatal Network in 2008–2014 (development set), using 12 easily collected perinatal variables. We used a cohort from 2015–2016 (N = 5810) as a test set. Among several machine learning methods we chose artificial Neural Networks (NN). The resulting predictor was compared with logistic regression models. In the test cohort, NN had a slightly better discrimination than logistic regression (P < 0.002). The differences were greater in subgroups of neonates (at various gestational age or birth weight intervals, singletons). Using a cutoff of death probability of 0.5, logistic regression misclassified 67/5810 neonates (1.2 percent) more than NN. In conclusion our study – the largest published so far – shows that even in this very simplified scenario, using only limited information available up to 5 minutes after birth, a NN approach had a small but significant advantage over current approaches. The software implementing the predictor is made freely available to the community.

## Introduction

In neonatology, as in other branches of medicine, there is the need to forecast the outcome of individual subjects based on their characteristics. Though comprising about 1 percent only of all births, very preterm infants contribute disproportionately to infant mortality, often representing more than half of it. Not surprisingly, they are the focus of several prospective cohort studies and ongoing databases which collect data for the purpose of benchmarking and quality improvement. Being able to forecast the survival probability of a small preterm infant would be important both for the individual infant (for instance, to better advise parents) and to allow a risk-adjustment between groups of infants (for instance, to compare the outcomes at different hospitals). Gestational age (GA) and birth weight are the most important predictors of survival in these infants. To refine predictions, other features are often considered, such as sex, mode of delivery, need for resuscitation or therapeutic procedures, values of physiological variables, etc. Current neonatal illness scoring systems such as CRIB^1^, CRIB-II^2^, SNAP^3^ etc., as well as the mathematical models on which they are built, have repeatedly been shown to be too imprecise to predict individual outcomes^4–7^, so they are not intended for clinical use in individual patients, but are instead used retrospectively to provide an overview of a population of patients, or to adjust for differences in baseline risk, to allow for objective comparisons when reporting patient outcomes in different populations.

At present, all models estimate probabilities of the outcome by fitting linear (mainly logistic) models to data. For example, the Vermont-Oxford Network uses a “risk-adjustment model” based on 7 characteristics to estimate the probability of in-hospital death^8^, which is recalculated yearly. While this approach has the advantage of being transparent and providing interpretable coefficients for each variable (in logistic regression, by estimating odds ratios of outcome for each 1-point increase of the value of the predictor), it is limited by the somewhat arbitrary model specification (and associated risk of misspecification) and by the reduced possibility of taking into account interactions between variables and non-linearities in the variable-outcome relationships.

In other branches of medicine newer methods, coming from the field of artificial intelligence rather than from statistics, have been tried and shown to outperform model-based methods in this endeavor^9^. Published experience with such methods in neonatology is however quite limited^10–12^, the results conflicting and they have failed to reach widespread use.

The aim of our study was to develop a state-of-the-art-machine learning approach to the prediction of survival of very preterm - very low birth weight infants in the Italian Neonatal Network, which we called the Preterm Infant Survival Assessment (PISA) predictor and to make it freely available. We aimed to test its predictive ability and compare it to other commonly used published logistic-based methods. To build the predictor, we chose easily and routinely collected variables (features), following the data collection protocol of the largest neonatal network worldwide (the Vermont-Oxford Network, www.vtoxford.org), to gain generalizability to other neonatal settings and ease of dissemination of our results.

## Methods

### Patients

The study population comprised neonates admitted to Italian hospitals participating in the Italian Neonatal Network (INN, www.neonatalnet.org) in 2008–2016. The INN is a voluntary collaboration of neonatal units across Italy, with the aim of coordinating national data collection for hospitals adhering to the Vermont Oxford Network (VON) and maintains an anonymized database comprising all infants born <30 weeks of gestation, as well as those with a birthweight of <1500 g, regardless of gestational age at birth, admitted to the participating hospitals [*N* = 80]. The database covered about 70 percent of all infants with the above characteristics born in Italy during the study period.

The whole database was divided into a development dataset (data from years 2008–2014), where models were trained and refined and a test dataset (data from years 2015–2016) where models were tested against new data as they became available.

In the development set, from the 27947 babies comprising the VON database for 2008–2014, we excluded infants born before 22 weeks [*N* = 30], because of only sporadic NICU admission at these early weeks and extremely high neonatal mortality. We also excluded infants with congenital anomalies from a pre-defined list [*N* = 1163]. We further excluded all cases with missing values for any predictor [*N* = 3007], leaving 23747 infants for analysis.

For the test set, we analyzed data for preterm infants from 2015 and 2016, with the same inclusion and exclusion criteria as before; in case of missing data, we excluded infants with missing birth weight or GA and retained infants with other missing values, filling the missing value with the “normal” (most frequent) value as suggested in other scoring systems^1,3^. The test dataset comprised 5810 infants.

Descriptive statistics for the development and test datasets are reported in Table 1. Before training, birth weight, GA and Apgar scores were conveniently normalized to have 0 mean and unit variance, to adjust for differences in scale that may slow down convergence of the learning algorithm. Furthermore, since values of the *race* variable were originally encoded as integers, they were encoded in a *1-of-k* fashion, to eliminate the implicit order dependency. The same preprocessing (but using mean and variance computed for the training set, in order to avoid bias) was applied to the test set.

**Table 1:** Descriptive statistics for the development (*N* = 23747) and test (*N* = 5810) datasets.

Characteristics	Levels	DEVELOPMENT		TEST	
		*n*	%	*n*	%
Birth weight (grams)	<1000	8801	37.06	2142	36.87
	1000–1500	14650	61.69	3616	62.24
	>1500	296	1.25	52	0.89
GA (weeks)	<26	3423	14.41	867	14.92
	26–30	12934	54.47	3204	55.15
	31–35	7218	30.40	1693	29.14
	>35	172	0.72	46	0.79
Apgar score (1 min.)	0–3	4206	17.71	950	16.35
	4–6	8355	35.18	2209	38.02
	7–10	11186	47.11	2651	45.63
Apgar score (5 min.)	0–3	760	3.20	149	2.56
	4–6	3096	13.04	754	12.98
	7–10	19891	83.76	4907	84.46
Sex	male	12006	50.56	2962	50.98
	female	11741	49.44	2848	49.02
Mode of Delivery	cesarean	19316	81.34	4689	80.71
	vaginal	4431	18.66	1121	19.29
Maternal race	black	1139	4.80	314	5.40
	hispanic	1561	6.57	374	6.44
	white	19705	82.98	4809	82.77
	asian	882	3.71	252	4.34
	other	460	1.94	61	1.05
Chorioamnionitis	no	20707	87.20	4951	85.22
	yes	3040	12.80	859	14.78
Prenatal care	no	1924	8.10	320	5.51
	yes	21823	91.90	5490	94.49
Antenatal steroids	no	4434	18.67	753	12.96
	yes	19313	81.33	5057	87.04
Maternal hypertension	no	17635	74.26	4365	75.13
	yes	6112	25.74	1445	24.87
Multiple birth	no	15780	66.45	3801	65.42
	yes	7967	33.55	2009	34.58
Died before discharge	no	20840	87.76	5147	88.59
	yes	2907	12.24	663	11.41

The development set refers to data from 2008 to 2014, the test set refers to data from 2015 to 2016.

The following variables were considered: gestational age at birth (completed weeks); birthweight in grams; Apgar scores at 1 and 5 minutes; sex; multiple gestation; mode of delivery; prenatal care; chorioamnionitis; maternal hypertension; race/ethnicity; antenatal steroids. All entities were defined per VON data collection protocol (www.vtoxford.org). We selected input variables based on their clinical significance and known relation with survival, as well as ease and accuracy of collection and presence in other illness severity scores already available. We considered variables only up to 5 minutes after birth, to minimize the effect of postnatal doctor-dependent decisions on survival estimations.

The endpoint of interest in this study was death before discharge from the NICU.

### Ethics, Consent and Permissions

This study was carried out as an analysis of the INN anonymised database in accordance with Italian law on observational research and was approved by the Ethics Committee of Azienda Ospedaliera “Ospedale di Lecco” on March 04, 2009, ref. 140109. Local Ethics Committees’ approval was also sought by all units participating in the study and parents provided informed consent to data collection, as required by Italian law. No protected healthcare information was collected.

The raw data analysed in this study are not publicly available due to stipulations about their use with participating hospitals, but may be available from the corresponding author on reasonable request and with permission of participating hospitals. The software implementing our method is made freely available to the scientific community, both as a web-service automating PISA score prediction (http://pisascore.itc.unipi.it) as well as the original source code (https://github.com/marcopodda/inn) used to train the models.

### Statistical and machine learning methods

The idea of the PISA score is to realize an effective predictor for preterm infant survival using a combination of a state of the art machine learning model together with a novel combination of perinatal input features upon which the prediction is computed.

As regards the first contribution, we have applied consolidated methodologies to identify the most suitable machine learning model for the task from a pool of candidate methodologies. Particular care has been taken with respect to the generalization performance of the model, i.e. the ability to correctly generalize the predictions of a trained model on samples not included in the data used for parameter fitting.

We have taken into consideration a pool of six state-of-the-art machine learning models, that are briefly reviewed in the following:

*Logistic Regression* (LR)^13^ is the baseline model in this study, widely used in a variety of fields, especially biostatistics. The key advantages of LR are its simplicity, the scalability to very large datasets and the interpretation it provides in terms of how unitary changes in an input feature influence the log-odds of the associated linear parameter. On the other hand, being a linear model, its predictive performances might be limited in presence of non-linear relationships in the data.

*k-Nearest Neighbor*^13^ is a memory-based model, where predictions are performed by similarity of the current sample to *k* nearest elements in the training set, according to the given distance metric. The key advantage of this method lies in its sheer simplicity, compensated by the difficulties in robustly determining the most appropriate similarity function as well as the choice of the *k* meta-parameter. Additionally, its computational complexity increases with the size of the training set.

*Random Forest* (RF)^14^ is a type of *ensemble methods* in which multiple learning models are combined together to improve generalization. The intuition behind ensembling is that a pool of simple models can yield better performances than a unique complex model, which might be more prone to overfitting due to its high variance. RF realizes an ensemble of decision trees, where a tree describes a decisional process such that at each node a branching decision is taken by confronting the value of one feature with a threshold. Both the structure of the tree and the thresholds are determined during the learning phase. The RF constructs multiple decision trees trained on random subsets of the training samples and of the data features, combining their predictions to provide the ensemble output. Decision trees have found wide application due to their simplicity and interpretability which, however, is typically lost in RF due to the high number of trees generated in the ensemble.

*Gradient Boosting Machine* (GBM)^15^ is another ensemble method combining a series of weak learners to obtain a stronger predictor. A GBM works by training a very simple base learner on all training samples, incrementally adding new learners to minimize the residual error. Typical choices for the learners are again the decision trees.

*Support Vector Machine* (SVM)^16^ is a supervised learning model built on the concept of a linear separator that is extended to deal with non-linear problems by exploiting the so-called *kernel trick*, that is an implicit map of the input vector into a high-dimensional feature space by means of a non-linear map induced by a kernel function. Currently, they are among the most widely used learning algorithms together with Neural Networks, because of their excellent predictive accuracies and sound generalization properties. On the other hand, their computational complexity can become exceedingly high for large scale problems and interpretability of the results of learning is often difficult. In this study, the SVM model used a Radial Basis Function (RBF) kernel.

*Artificial Neural Networks* (NNs)^17^ are a machine learning model loosely inspired by a computational view of the brain and neuron organization. The key idea is that neurons can be thought of as computational units that acquire signals from nearby neurons through synaptic connections and are activated if the accumulated signal strength exceeds a certain threshold. Over the years, NNs have developed in increasingly complex and powerful architectures and algorithms which have lead to the recent Deep Learning^18^ revolution. Neural models are very popular due to their effectiveness in a variety of applications, which is the result of their ability in representing complex, non-linear decision boundaries, coupled with manageable computational costs (also thanks to parallel implementations). As for the SVM model, however, interpretability of the results is still problematic, although some works are beginning to address the problem^19^.

The rationale behind this selection of candidate models was to provide a reasonable coverage of different methodologies, characterized by different inductive biases, interpretability as well as computational costs. Also, we have taken into consideration the availability of consolidated, trustable and open-source implementations of the models.

### Metrics

In order to compare different methods which do not provide a yes/no answer, but estimate instead the probability of survival rather than exact outcome prediction, we employed the *Area Under the Receiver Operating Characteristics Curve* (AUROC) as the metric of choice. The AUROC provides a measure of the model’s ability to discriminate between those with and without the outcome. A value of 0.5 indicates that the model is equivalent to a coin toss, while a value of 1 indicates perfect prediction^20^. As a method could provide an overall good prediction over the entire sample, but could behave badly for some infants, we also assessed the goodness of fit of all the considered models by calculating the *Brier loss*^21^. The higher the Brier loss, the worse is the goodness-of-fit, with values 0.25 equivalent to a coin toss and of 0 for a perfect forecast.

### Model Selection

We selected the best possible model among the candidate methodologies with hyper-parameter optimization. Hyper-parameters are a set of additional, model-dependent parameters that are not inferred automatically by the learning algorithm but need to be specified before the learning phase: a common example of hyper-parameter is the value of *k* in k-Nearest Neighbor or the number of hidden units in a Neural Network. Hence, finding sub-optimal values of the hyper-parameters is crucial to ensure proper generalization. The hyper-parameter optimization procedure, which was repeated separately for each candidate learning methodology, encompassed the following steps: (*a*) Firstly, a set of suitable hyper-parameters to optimize was identified; for each of them, a range of candidate values was specified. These choices are dependent both on our expertise and on the computational cost needed to train the models. (*b*) Secondly, a predictor was learned for all the possible combinations of hyper-parameters and its out-of-sample performance was estimated using 5-fold Cross Validation (CV), i.e. we trained the model with a fixed combination of hyper-parameters using 5 different realizations of the development data (2008–2014), each one accounting for 80% of the total training set size and validated its performance in the remaining 20%. The resulting 5 AUROC scores were averaged to produce the final CV score. This phase yielded 6 models with optimized hyper-parameters, one for each learning methodology. (*c*) Finally, the model with the highest AUROC mean was chosen and trained once again with all the available training data, to produce the final predictor. As a side note, we report that CV was designed to preserve the stratification of the outcomes, i.e. the proportion of deaths in each training/validation fold was as close as possible to the one of the entire development set. All the model selection phase has been implemented using Python’s *scikit-learn* library^22^.

### Model Evaluation

Once the final predictor was trained, we assessed its performances on the external test set (2015–2016). These test data have been acquired at a later time than the development set and have not entered the model selection phase at any stage. This is a best practice which is adopted in order to not overestimate the actual generalization capability of a model. We contrasted the performances of the final predictor against a selection of LR probabilistic classifiers for preterm infants survival, either commonly used as baselines or found in literature.

More in detail, we have compared with the following:

- A baseline predictor built with birth weight as the only feature (BW);
- A baseline predictor built with birth weight and GA as features (BW + GA);
- A predictor for very preterm infants developed by Manktelow *et al*.^23^ (Manktelow);
- A predictor for extremely preterm infants developed by the National Institute of Child Health and Human Development^24^, USA (Tyson);
- A predictor built using the same features as a model used by VON for risk-adjustment purposes^8^ (Logistic1), which has been validated also on an Italian sample^25^;
- A predictor built using the same features as the PISA predictor (Logistic2): GA, Birth weight, Apgar scores at 1 and 5 minutes, sex, mode of delivery, maternal race/ethnicity, chorioamnionitis, prenatal care, antenatal steroids, maternal hypertension, multiple birth.
  Models BW, BW + GA, Logistic1 and Logistic2 have been trained with the same development set and model selection procedure described above; as regards models Manktelow and Tyson, we utilized the regression coefficients published in their respective papers and estimated their performance directly on the test data. Note that models Manktelow and Tyson are based upon specific sub-populations rather than the more heterogeneous population that our data represents and are therefore considered highly specialized in predicting the survival of infants belonging to a specific cohort of newborns. To give a wider and fairer comparison, we analyzed the predictions obtained on:
- The entire INN test set [*N* = 5810];
- A subset [*N* = 842] of infants born up until the 25th gestational week whose birth weight is between 400 and 999 grams (ELBWI, Extremely Low Birth Weight Infants);
- A subset [*N* = 3616] of infants whose birth weight is between 1000 and 1500 grams (VLBWI, Very Low Birth Weight Infants);
- A subset [*N* = 3378] of singleton infants born within the 23rd and the 32nd gestational week (singletons).

In particular, Manktelow is specialized in predicting infants from the SINGLETONS subgroup, while Tyson is specialized in predicting infants from the ELBWI subgroup. Additionally, we analyzed performances on the VLBWI subgroup, as it is a commonly examined sub-population in literature.

## Results

### Model Selection

The results of the model selection phase are summarized in Table 2. The LR model displays an excellent adherence between training and CV performances, although its performance is relatively poor with respect to models able to represent non-linear interactions such as SVM and NN; a similar argument is applicable to k-Nearest Neighbor. The RF model displays a wide spread between training and CV performance, which however leads to a concrete improvement in CV. GBM, SVM and NN models have quite similar performances both in training and CV; close agreement between the training and CV performance indicates that they most likely did not overfit the training set. Overall, the best performing model is the NN, although its performance is closely followed by that of SVM and GBM. Since the purpose of this first phase is only to select *one* best model to be used for final testing, it is of little relevance if the difference between NN and the runner-up models is statistically significant. Therefore, we chose the NN as the reference model and retrained it using the entire INN development data, as described in the methods section. The resulting model is the PISA predictor.

**Table 2:** Results of the model selection procedure on the INN development set [*N* = 23758].

MODELS	TRAINING	CROSS-VALIDATION
Logistic Regression	0.9105 ± 0.0010	0.9098 ± 0.0038
K-Nearest Neighbor	0.9142 ± 0.0012	0.9108 ± 0.0040
Random Forest	0.9373 ± 0.0010	0.9138 ± 0.0053
Gradient Boosting Machine	0.9200 ± 0.0011	0.9147 ± 0.0048
Support Vector Machine	0.9170 ± 0.0013	0.9147 ± 0.0047
**Neural Network**	0.9171 ± 0.0010	**0.9149** ± **0.0047**

For each candidate model, its training and validation AUROC (averaged over 5 CV iterations, ±standard deviation) is reported. The selected model is highlighted in bold.

### Model Evaluation

Table 3 summarizes the AUROC of the evaluated predictors in the INN test set (2015–2016) with respect to the various subgroups described previously. Not unexpectedly, all models performed better when the ranges of weights and GA weeks were larger and performed worse when predicting infants in the ELBWI and VLBWI cohorts. For all subsets of data, as well as for the full dataset, the PISA predictor outperformed all other prediction models. On the full test set, the improvement in AUROC is 0.6% with respect to the second-best scoring model (P < 0.002, according to Delong’s test for statistical significance of the difference between dependent ROC curves). The PISA predictor achieves the greatest improvement in the most “difficult” subset (VLBWI): in fact, it improves the AUROC by 3.62% with respect to the second-best scoring model. It also performs remarkably better than specialized models: in the ELBWI case, it improves the AUROC by 10.10% with respect to Tyson; in the SINGLETONS case, it improves the AUROC by 8.62% with respect to Manktelow. Furthermore, it appears that the variables taken into account in this study improve discrimination: in fact, the two best-scoring models (PISA and Logistic2) are the ones that use the proposed set of covariates.

**Table 3:** AUROC values of different preterm survival predictors on the 2015–2016 test data under different segmentations of the original population.

	BW	BW + GA	Manktelow^23^	Tyson^24^	Logistic1	Logistic2	PISA
FULL [*N* = 5810]	0.8733	0.8875	0.8643	0.8738	0.9023	0.9081	**0.9136**
ELBWI [*N* = 842]	0.6943	0.7233	0.6901	0.7076	0.7584	0.7725	**0.7791**
VLBWI [*N* = 3616]	0.6761	0.7156	0.6871	0.6703	0.7878	0.7992	**0.8281**
SINGLETONS [*N* = 3378]	0.8319	0.8471	0.8165	0.8322	0.8733	0.8802	**0.8869**

The conditions listed in the leftmost column indicate the inclusion criteria for subjects on which the AUROC is computed. *N* indicates the number of test subjects that meet the inclusion criteria. ELBWI: <26 weeks and 400–999 g; VLBWI: 1000–1500 g. SINGLETONS: singletons with 23 ≤ GA ≤ 32. BW: birth weight; GA: gestational age.

**Figure 1:**
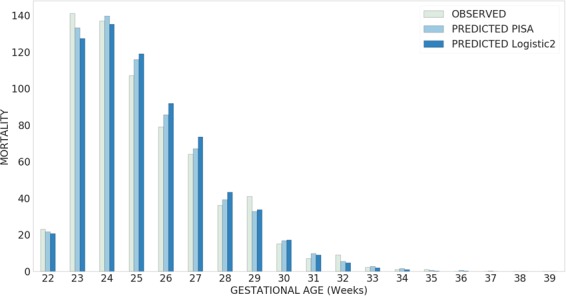
Plot of the observed mortality vs. mortality predicted by the two best-scoring models (PISA and Logistic2), per gestational week, in the test dataset (2015–2016).

Figure 1 shows the predicted number of deaths at each gestational week, compared with observed number of deaths. The PISA predictor was better than the Logistic model in the most important weeks (from 22 to 29 weeks, where about 95% of deaths occurred). In these weeks, 628 death were observed, versus 635.1 as predicted by the PISA algorithm and 644.5 predicted by the logistic model.

Using a cut-off of death probability of 0.5, the PISA predictor misclassified 67/5810 (1.2 percent) less than the LR model.

Table 4 reports the goodness of fit achieved by the considered models. Although the difference with the Logistic2 model appears to be marginal, the PISA predictor achieves the best goodness of fit in every considered scenario. In general, model fit is acceptable with the exception of the ELBWI subgroup, where some of the models (BW, BW + GA, Manktelow) obtain a Brier score worse than the one of a random classifier. Perhaps not surprisingly, the best goodness of fit overall is obtained in the VLBWI subgroup, as VLBW infants comprise the largest proportion of the cohort and the model developed will rely heavily on this group of infants compared to others. Note that on this infants subgroup the models tend to provide very sharp outputs (i.e., predicted probabilities are close either to 1 or 0) in case of a correct prediction, while being poorly selective, i.e. predicting probabilities close to the decision threshold but still incorrect, in case of a prediction error.

**Table 4:** Brier loss of the examined predictors on the 2015–2016 test data under different segmentations of the original population.

	BW	BW + GA	Manktelow^23^	Tyson^24^	Logistic1	Logistic2	PISA
FULL [*N* = 5810]	0.1001	0.0866	0.0812	0.0692	0.0640	0.0623	**0.0612**
ELBWI [*N* = 842]	0.3588	0.2997	0.2722	0.2174	0.1980	0.1912	**0.1892**
VLBWI [*N* = 3616]	0.0316	0.0265	0.0219	0.0220	0.0209	0.0201	**0.0197**
SINGLETONS [*N* = 3378]	0.1145	0.1019	0.0982	0.0872	0.0797	0.0768	**0.0757**

Lower values indicate better goodness of fit.

## Discussion

This study demonstrates that a machine learning approach predicts survival in very preterm neonates better than logistic models and allows a more refined approach to risk-adjustment, a prerequisite to obtain unbiased comparisons between groups and carry out observational studies or quality-improvement initiatives. This result is not surprising, as machine learning methods have been shown to outperform logistic methods in several fields of medicine^9^.

Using variables collected up to birth (the first 5 minutes), the PISA predictor, as well as the Logistic model, showed an AUROC of over 0.9. Care must be taken not to misunderstand this figure. An AUROC of 0.9 at birth does not mean that 90 percent of the risk of dying is already defined at birth, so that subsequent clinical care can only influence the remaining 10 percent. With a total mortality rate of 12 percent as in this sample, a “blind” *a priori* forecast of survival for all infants would end in a correct estimate in 88 percent of cases, but an AUROC of 0.5 only. In fact, a ROC area represents the probability that the score of a randomly selected infant who died will be higher than the score in of a randomly selected surviving infant. Thus, even a ROC area of 0.9 would not look so impressive, meaning a 10 percent overlapping of scores between deaths and survivals. Moreover, this tells nothing about the calibration of the model: it only tells that the system can correctly predict (in 90 percent of cases), among 2 random subjects with different outcome, who will likely survive.

The problem of forecasting the survival of very preterm infants has a long history. In this population, the risk of dying is mainly driven by immaturity of organs and functions, as measured especially by low GA and birth weight. This is shown in our study by the high AUROC brought about by the LR model with only birth weight and GA, which is only 2.5 percentage points less discriminative than the full PISA predictor. The clinical experience is however that even when GA and birth weight are considered, there remains a variability in outcomes. To tackle this problem, 2 main approaches have been used. The first uses physiological or clinical variables (such as the degree of acidemia soon after birth or in the first 12–24 h, or need for resuscitation, ventilation; or the presence of physiologic disturbances such as oliguria; or pathological conditions such as seizures, etc)^1–3^. This first approach has led to the construction of clinical scores, where the coefficients obtained from statistical models are transformed into scores, which are summated. The higher the score, the higher the mortality risk. These scores have however proven to be too unreliable to guide decisions in individual infants, require the collection of ad hoc features and - given that the time window of observation extends to 12–24 h - are also influenced by doctors’ decisions, i.e. they compound the baseline risk of the infant with subsequent clinical management. On the other hand, fully physiology based prediction models like TRIPS^26^ and TRIPS-II^27^, which can be sequentially measured over time, allow to provide updated predictions of mortality and morbidity as the infants’ conditions change.

Another approach instead uses richer demographic and obstetrical information to estimate the baseline risk of the infant^8,28^. In this case, instead of a summated score, a prediction based on equations is obtained. This approach is often used a posteriori, as it requires computations difficult to carry out at the bedside and is used to average as best as possible the baseline risk of groups of infants, with the purpose of comparing groups of infants, not individual babies. In this case, generally the time frame of data collection stops at birth or at the delivery room and is less influenced by subsequent clinical decisions and management, but usually loses the information on clinical conditions of the infant. Our study clearly belongs to this second case, using a machine learning method to ascertain the risk. Among several machine learning methods, NN had marginally better performance than others. To the best of our knowledge, only few previous attempts at using machine-learning methods have been published, all using NN as we did. Zernikow *et al*.^10^, in a single-center study on infants <32 weeks or <1500 g, found a better performance of NN in predicting death than LR. They used several (29, of which 2 prenatal and 27 perinatal) variables, some of which potentially influenced by the doctor’s opinion (e.g. “condition on admission”), which could be difficult to generalize across hospitals. Ambanavalan *et al*., using again NN vs. logistic regression models, found instead a similar performance in ELBW infants in a single center^11^. Subsequently, this Author, in a large multicenter study based on the National Institute of Child Health and Human Development Neonatal Research Network on ELBWI (5800 infants in the training set and 2500 in the test set)^12^, confirmed that NN was not superior to LR in predicting death. These authors tested the 2 methods under a variety of scenarios (limited prenatal data; full prenatal data; first 5 minutes of life, first 24 hours of life; first 7 days of life) and found that the better prediction was obtained using the information up to 5 minutes of life, as in our study.

The reason for different results in these studies (and ours) is not clear, but samples studied differed for several key variables, including GA span, birth weight, presence/absence of congenital anomalies which - as the results in subsamples in our study clearly show - influence results; the variables entered in the model were partly different; and the NN methods might have been different too.

In our study, the comparison between the logistic model and the PISA algorithm is of particular interest. Both make use of the same features and the difference in performance between them is due to the ability of NN to take into account interactions between variables and non-linearities in variable-outcome relationships. Logistic regression models (as well as other linear models) are easily understood by clinicians and their results are easily translated in measures of individual risk (Odds ratios). Yet this apparent ease of interpretations is lost when interactions and effect modifications are present, especially when more than one interaction is present. Interactions between risk factors on outcomes such as mortality or morbidity are not rare, as some recent studies have demonstrated, e.g. between GA and chorioamnionitis/pre-eclampsia^29^, or GA and being small-for-GA^30^, or steroid prophylaxis and multiplicity^31^.

The NN improved the predictive performance of less than 1 percentage point over the LR using the same features in the full sample, but the performance of NN remained much higher in subsamples such as infants between 1000 and 1500 g. Moreover, the predicted number of infants dying was much closer to the observed number for the PISA algorithm than for the logistic regression, both for the whole sample and for the subsample from 22 to 29 weeks.

It is likely that a ceiling effect is present, i.e., basal conditions of infants do not allow to predict much further mortality, as some of the deaths are “unexplained” by factors that can be measured at birth, including clinical course, therapy and late occurring events. In fact, in these infants most of the deaths occur in the first few days after birth and the risk factors identified in this and other studies (e.g. GA, low Apgar score, low birth weight, etc) mostly act on early deaths, whereas their effect on late deaths could be less direct. Pepe *et al*.^32^ showed that for any risk factor to influence substantially prediction, its odds ratio must be very high. Odds ratios for several of these established risk factors (such as gender, or steroid prophylaxis receipt, or multiple birth) are instead below 2: a value which - though important when applied to large numbers of infants - is too low to meaningfully influence prediction in individuals. Thus, it must be acknowledged that even the PISA predictor, like all other model-based methods, is still too imprecise to be used for predicting an individual infant’s outcome.

This study had several strengths. It is the largest study published so far (more than 23700 infants in the training set, 5800 in the test set). Additionally, we used completely different samples for developing the PISA algorithm and testing it. Often, to obviate the need of very large samples, this requirement is relaxed and other methods are used such as splitting the same sample into two subsamples, or using a leave-one-out method. However, testing a model on the same sample where it was generated spuriously appears to increase discrimination.

Moreover, this was a multicenter study enrolling infants from the large majority of units in Italy. A multicenter data collection, hence inter-hospital variability in clinical practices, while probably reducing overall discrimination, avoids the possible spurious associations of a variable with outcome due to single-centers idiosyncrasies and should ensure a greater generalizability of our results. All hospitals used the same protocol for data collection (that of the VON), which ensures both reproducibility and ease of data collection and transferability of results to other hospitals and settings.

This study also had weaknesses. Our data collection lacked detail - as is often the case for large epidemiologic studies - and most of the predictors were collected as binary variables (present/absent), thus lacking severity details. This should however have limited our forecasting ability, so that our results are conservative. Moreover, we were limited to the set of variables collected and we have no evidence that by using other variables the results would not change. Our database did not include information allowing to compute illness severity scores such as CRIB or SNAPPE, so we cannot comment on the performance of the PISA predictor in comparison with them. Nevertheless, other studies have shown that logistic methods - tested in the present study and found inferior to PISA - are more accurate than such scores^8,25^.

In conclusion, however, this study shows that ML methods can provide better discrimination than LR models in this problem, especially concerning goodness of fit on most critical groups. As the software implementing our method is made freely available to the scientific community, both as a web-service automating PISA score prediction as well as the original source code (see “Methods” for details) to train the models, we await confirmation of our results in other samples from other settings.

## Electronic supplementary material

Supplementary Information
