# Supplementary material for: A machine learning approach to estimating preterm infants survival: development of the Preterm Infants Survival Assessment (PISA) predictor
Source: Sci Rep. 2018 Sep 13;8:13743. doi: 10.1038/s41598-018-31920-6 (PMC6137213; doi:10.1038/s41598-018-31920-6)
Supplement: Supplementary file 1 — Supplementary Information [file 41598_2018_31920_MOESM1_ESM.pdf]

A machine learning approach to estimating preterm infants survival: development of the Preterm Infants Survival Assessment (PISA) predictor

Marco Podda, Davide Bacciu, Alessio Micheli,
Roberto Bellù, Giulia Placidi, Luigi Gagliardi

Supplementary Information: list of Italian Neonatal Network Members

Members of the Italian Neonatal Network include Gianpaolo Mirri, Manuela Condò, Daniela Turoli, Rinaldo Zanini (Lecco); Mara Vanzati, Fabio Mosca (Fondazione IRCCS Ca' Granda Ospedale Maggiore Policlinico, Università degli Studi di Milano); Giuseppe De Nisi, Paola Polacco (Trento); Elena Villa, Mario Barbarini (Como S. Anna); Valeria Fasolato, Caterina Franco (Milano Macedonio Melloni); Raffaella Contiero (Ferrara); Serena Ellero, Luigi Cattarossi (Udine); Laura Abbiati, Cesarina Borroni (Monza); Giovanna Prandi, Claudio Fabris, Francesca Vielmi, Silvia Borgione (Torino S.Anna); Massimo Agosti, Francesco Tandoi, Rosanna Guidali (Varese); Mario De Curtis, Claudio Tozzi, Renato Lucchini (Roma Umberto I); Marina Battaglioli, Gian Luca Lista, Paola Introvini (Milano Buzzi); Fabrizio Ferrari, Claudio Gallo (Modena); Elvira Bellante (Palermo Cervello); Chiara Bottura, Aurelija Zeringyte, Francesca Pasquali, Simona Boccacci (Mantova); Giuseppe Latini, Raffaella Giannuzzi (Brindisi); Stefano Martinelli, Alberto Brunelli (Milano Niguarda); Maria Lucia Di Nunzio, Antonio Vendemmiati (Campobasso); Giovanna Carli, Michela Alfiero Bordigato, Marco Filippone, Davide Meneghesso (Camposampiero); Nicola Romeo, Palma Mammoliti (Rimini); Emanuele Mastretta, Laura Barberis, Daniele Farina (Torino); Gianpaolo Gancia, Cristina Dalmazzo (Cuneo); Marcello Napolitano, Francesco Messina (Napoli Villa Betania); Rosario Magaldi, Matteo Rinaldi, Rosangela Litta (Foggia); Paola Lago, Vincenzo Zanardo, Lino Chiandetti (Padova); Stefano Visentin (Treviso); Giuseppe Presta, Davide Cella (Tricase); Carlo Poggiani, Daniela Ferrari, Sara Parati (Cremona); Francesco Lombardo, Rosetta Grigorio (Siracusa); Graziano Barera, Maddalena Bove, Antonella Poloniato (Milano San Raffaele); Giampiero Burgio, Elena Sala (Bergamo); Ignazio Barberi ,Venera Tiralongo, Alessandro Arco, danila Mazzeo (Messina); Carlo Dani, Simone Pratesi (Firenze Careggi); Valeria Mignatti, Gina Ancora,

Giacomo Faldella, Sara Grandi, Chiara Locatelli (Bologna S.Orsola); Mauro Stronati, Gianfranco Perotti (Pavia); Gaetano Chirico, Claudio Migliori (Brescia); Sergio De Marini, Vincenzo Forleo (Trieste); Roberto Paludetto, Letizia Capasso, Giuseppina Mansi, Francesco Raimondi (Napoli Federico II); Gianni Bona, Ilaria Stucchi, Silvia Savastio, Federica Ferrero, Andrea Parola (Novara); Ezio Maria Padovani, Elena Viviani, Laura Pecoraro (Verona Borgo Trento); Rocco Agostino , Camilla Gizzi, Luca Massenzi (Roma Fatebenefratelli Isola Tiberina); Hubert Messner, Alex Staffler (Bolzano); Gennaro Salvia (Napoli Buon Consiglio - F.B.F.); Luigi Esposito, Vincenzo Forziati, Giuseppe Latorre (Acquaviva delle Fonti); Fabrizio Sandri, Stefania Alati, Fabrizio Demarca (Bologna Ospedale Maggiore); Oreste Lombardi, Carmine Deni Costabile, Gianfranco Scarpelli (Cosenza); Claudio Cavalli, Enrico Volante, Sabrina Moretti (Parma); Olga Ganguzza, Bartolomeo Spinella (Palermo Buccheri La Ferla F.B.F.); Cristina Haass, Eleonora Scapillati, Chiara Consigli (Roma S. Pietro F.B.F.); Alberto Gatta, Pasqua Quitadamo (S.Giovanni Rotondo); Antonio Boldrini, Marco Vuerich, Emilio Sigali (Pisa); Patrizio Fiorini, Letizia Petrucci, Marco Moroni (Firenze Meyer); Patrizia Bragetti, Paola Casucci, Liliana Minelli, Daniele Mezzetti (Perugia); Luigi Orfeo, Maria Gabriella De Luca (Benevento); Nicola Laforgia, Alessandro Grassi (Bari Policlinico); Andrea Dotta, Ferdinando Savignoni (Roma Bambino Gesù); Franco Bagnoli, Claudio De Felice, Silvia Badii (Siena); Augusto Biasini, Antonio Belluzzi, Marcello Stella (Cesena); Costantino Romagnoli, Enrico Zecca, Giovanni Barone (Roma Policlinico Gemelli); Paolo Colleselli , Luca Vecchiato, Silvia Nicolussi (Vicenza); Paolo Giliberti, Giovanni Chello, Silvana Rojo, Massimiliano De Vivo (Napoli Monaldi); Chiara Giovanettoni, Clelia A. Colnaghi, Valeria Manfredini (Rho); Elena Verucci, Giulia Placidi, Luigi Gagliardi (Versilia Viareggio); Cesare Belloni, Giuseppe Carrera, Chiara Zambetti (Lodi); Paolo Biban, Alessandra Serra, Francesco Sacco (Verona Ospedale Civile Maggiore); Gennaro Vetrano, Giuseppe Furcolo, Brigida Pasquariello (Benevento Ospedale Fatebenefratelli); Luigi Falco, Gaetano Ausanio, Italo Bernardo, Antonella Capasso (Caserta); Giuseppe Marchesano, Norberto Nosari, Paola Sarnelli (Nocera Inferiore); Giovanni Ciraci, Daniele Merazzi (Como Valduce); Diego Gazzolo, Francesca Temperini, Miriam Sabatini, Micaela Colivicchi (Alessandria); Antonio Del Vecchio, Marzia Tarantino (Bari Di Venere); Giancarlo Gargano, Simona Pedori (Reggio Emilia); Massimo Bellettato, Roberta Pesavento, Alessandra Cesaro, Massimo Scollo (Thiene); Isabella Mondello (Reggio Calabria); Aniello Pugliese, Claudio Iervolino (Napoli Cardarelli); Giovanni Corsello, Mario Giuffré (Palermo Policlinico Giaccone); Pasqua Betta, Mario Giuseppe Romeo, Alessandro Saporito (Catania Policlinico Rodoligo); Maria Grazia Leone, Alessandro Rodonò (Catania Ospedale Santo Bambino); Alessia Franceschi, Francesco M. Risso (Genova Gaslini); Mauro Carpentieri, teresa Vecchiano, Maria Pia Cigliano (Napoli SS Annunziata); Piermichele Paolillo, Simonetta Picone (Roma Casilino); Antonio Marra, Gessica Rossetti, Tiziana Testa (Moncalieri); Fernando Del Cuore (Lecce); Francesco Crescenzi (Terni); Gianna Poloni, Maria Chiara Russo (Torino Maria Vittoria); Francesco Nigro, Gabriella Lucia Tina (Catania Ospedale Garibaldi); Patrizia Brindisino (Chieti); Raffaele Gurrado (Ospedale SS.

Annunziata); Maurizio Felice, Isabella Formica (Ospedale Bolognini Seriate).
